# Chromosome-scale assembly of apple mint (*Mentha suaveolens*)

**DOI:** 10.1186/s12863-024-01278-y

**Published:** 2024-11-08

**Authors:** Alana Firl, Meric C. Lieberman, Nestor Kippes, Helen Tsai, Eric Dowd, Luca Comai, Isabelle M. Henry

**Affiliations:** 1 Department of Plant Biology and Genome Center, University of California, Davis, CA 95616 USA; 2 Mars-Wrigley Plant Sciences Hub, Davis, CA 95616 USA; 3 Nucicer Inc, Davis, CA 95618 USA; 4 Ingredient Science, Mars Wrigley, 1132 W. Blackhawk St, Chicago, IL 60642 USA

**Keywords:** Genome sequencing, Mint, Mentha, Chromosome, Oil, Genome assembly

## Abstract

**Objectives:**

Mint oil is used in various commercial applications world-wide. Mint oil is typically harvested from commercial clones of peppermint or spearmints. Spearmints are the product of a cross between two diploid species: *Mentha longifolia* (horse mint) and *Mentha suaveolens* (apple mint). Peppermints are the product of an additional hybridization step between spearmint and an octoploid *Mentha aquatica* (water mint). Here, we present a chromosome-scale assembly of the genome of a clone of *M. suaveolens*. Together with the previously assembled genome of *M. longifolia*, these assemblies are instrumental in addressing questions regarding the origins of spearmint and peppermint oil and the genomic composition of commercial spearmints, and to start elaborating strategies for mint cultivar improvement.

**Data description:**

A Falcon assembly of the genome of *M. suaveolens* was generated from 103X coverage of PacBio long reads. Additional scaffolding was conducted by Dovetail Genomics, using a Chicago library, and a HiC library. The resulting assembly had an N50 of 44.7 Mb, and 98.45% of the 536 Mb of the assembly were contained within 12 large superscaffolds. Finally, a genetic map was applied to correct persistent misjoins. Illumina RNA-Seq libraries from a variety of tissues were used to annotate the genome.

## Objective

Mint oils are essential oils with a wide variety of commercial applications. Mint oil is produced in the glandular trichomes present on the leaves of plants from the genus Mentha and different clones vary widely in the type of oil they produce. The two main oil types are spearmint oil and peppermint oil. Spearmint oil is characterized by a sweeter note and usually contains a high percentage of carvone. Peppermint oil provides a cooling sensation, mostly associated with the presence of menthol, which interacts with thermoreceptors. Mint oil is a complex mix of many different compounds and it is difficult, if not impossible, to recreate it artificially. Commercial mint plants are clonally propagated and many of the current commercial clones are decades old. Mint improvement is needed in order to address threats such as stagnant yields and disease pressure. Genomic tools, such as reference genomes for the commercial clones and their parental species will be instrumental in facilitating these efforts.

Spearmint clones originate from a hybridization event between two diploid parental species called *Mentha suaveolens* (apple mint) and *Mentha longifolia* (horsemint). A genome assembly for horsemint was previously published [1, 2] but no reference genome is available for apple mint. Here we present the reference genome for a clone of apple mint. This data was collected and developed in the context of a broader project aimed at understanding the relationship between the different Mentha genome and, specifically, understanding the genomic constitution of the commercial mints (spearmint and peppermint) in relationship to their parental genomes. Apple mints are used as ornamentals and as cooking herbs but are typically not grown commercially. Characterizing their genome is nonetheless critical because of their role in the formation of the cultivated mints traits.

## Data description

A Falcon assembly was generated from 103X coverage of PacBio long reads. Additional scaffolding was conducted by Dovetail Genomics, using a Chicago library, followed by a HiC library (Table 1). When the two scaffolding approaches were in conflict, the HiC library was prioritized. Finally, a genetic map was applied to correct persistent misjoins [3]. The resulting genomic reference included 12 chromosome scaffolds with an N50 of 47 Mb, and overall size of 526 Mb (Table 1). This genomic assembly was analyzed using the BUSCO tool [4, 5], reporting 97.8% complete, 0.2% fragmented, and 2.0% missing BUSCO(s) for the 2,326 BUSCO groups in the eudicots_odb10 dataset.

For genome annotation, 631.8 million Illumina RNASeq reads (Table 1) were obtained from normal and / or water-stressed conditions for the following tissue types: stem, root, flower (buds, young, and old) and leaf (young, mature, senescent). After initial demultiplexing and quality control, RNASeq libraries were mapped to the genomic assembly using hisat2 [6], and the hisat2 output mapping bam files were used as input for the Braker2 annotation software [7]. Braker2 generated a CDS annotation containing 111,426 unique transcripts, representing a coding space of 112 Mb. Functional annotation was obtained using BioBam’s Omicsbox on the Braker2 coding sequence reference. This process included blasting to NCBI’s NR database, protein mapping using InterProScan, and functional analysis GO Annotation mapping. The resulting annotated coding sequence reference includes 62,995 transcripts with functional annotation data, and 28,866 with no associated data. The genomic reference was annotated using the EDTA transposable element pipeline to highlight LTR, TIR, Helitron, and other element types [8].

**Table 1:** Overview of data files/data sets

Label	Name of data file/data set	File types (file extension)	Data repository and identifier (DOI or accession number)
*Data file 1*	Raw Hi-C reads	*Fastq file (.fq)*	NCBI SRA Accession number SRP507005 https://identifiers.org/ncbi/insdc.sra:SRP507005 [9]
*Data file 2*	Raw Chicago reads	*Fastq file (.fq)*	NCBI SRA Accession number SRP507005 https://identifiers.org/ncbi/insdc.sra:SRP507005 [9]
*Data file 3*	Assembled genome	*Fasta file (.fa)*	Figshare: 10.6084/m9.figshare.26938906.v1 [10]
*Data file 4*	Predicted genes	*Gff3 file (.gff3)*	Figshare: 10.6084/m9.figshare.26938906.v1 [10]
*Data file 5*	Predicted genes – CDS	*Fasta file (.fa)*	Figshare: 10.6084/m9.figshare.26938906.v1 [8]
*Data file 6*	Predicted repetitive sequences	*Gff3 file (.gff3)*	Figshare: 10.6084/m9.figshare.26938906.v1 [10]
*Data file 7*	Assembled genome	*Fasta file (.fa)*	NCBI GenBank Genome Assembly ID GCA_041501505.1 https://identifiers.org/ncbi/insdc.gca:GCA_041501505.1 [11]
*Data set 1*	Raw RNA-seq reads	*Fastq file (.fq)*	NCBI SRA Accession number SRP507005 https://identifiers.org/ncbi/insdc.sra:SRP507005 [9]
*Data set 2*	Raw PacBio reads	*PacBio basecall format (bax.h5)*	NCBI SRA Accession number SRP507005 https://identifiers.org/ncbi/insdc.sra:SRP507005 [9]

### Limitations

The assembly presented here is the first genome assembly undertaken as part of a wider mint genomic project and it was developed several years ago (starting in 2016), when several approaches to genome assembly were being developed in parallel and an optimal pipeline was not yet defined. Therefore, we employed different technologies (PacBio long-reads, Bionano Optical Mapping, Chicago and Dovetail libraries and a genetic map) in an arbitrary order. A different order might have generated a better final assembly. In the end, we benefited from a genetic map which was overall in very good agreement with our assembly and provided good support for our approach.

Some of the data gathered as part of this project is now relatively old and the technologies have improved significantly since then, with lower error rates and longer reads. For example, the PacBio long-reads were not of HiFi quality.

The annotation was based on RNA-Seq reads from a variety of tissue types but it is of course possible that some genes were not expressed in any of these tissue types. Of particular importance to mint oil is gene expression in the glandular trichomes. We did not include expression data from glandular trichomes specifically because of the difficulty in extracting them. We expect that many of the genes expressed in the glandular trichomes will be represented in the leaf extracts but dilution with RNA from other cell types may hinder detection of genes expressed at low level.

In addition, mint oil composition can change drastically as the plants develop [12]. Therefore, we expect that gene expression profiles of leaves and glandular trichomes will change over time. Here, we included leaves at three different stages, which we expect will represent most of the transcripts involved in oil biosynthesis but probably not all.

Finally, the genome of clone sequences is diploid and highly heterozygous. Some of the genetic variation present within this clone was there not captured in our consensus assembly.

## Data Availability

The data described in this Data note can be freely and openly accessed at the National Center for Biotechnology Information Short Read Archive [9] and FigShare [10] as described in Table 1. The available datasets are summarized in Table 1. The *Mentha suaveolens* clone used in the study, *Mentha suaveolens Ehrh. subsp. suaveolens* (Pl 557898 or CMEN 13), can be obtained from the USDA Mint Germplasm Collection (Corvallis, OR, USA).

## Abbreviations

HiFi: High-fidelity
BUSCO: Benchmarking Universal Single-Copy Orthologs

## References

[1] Vining KJ, Pandelova I, Lange I, Parrish AN, Lefors A, Kronmiller B, et al. Chromosome-level genome assembly of *Mentha longifolia* L. reveals gene organization underlying disease resistance and essential oil traits. G3. 2022;12(8):jkac112. 10.1093/g3journal/jkac112 10.1093/g3journal/jkac112 PMC9339296 35551385

[2] Vining KJ, Johnson SR, Ahkami A, Lange I, Parrish AN, Trapp SC, et al. Draft genome sequence of *Mentha longifolia* and development of resources for Mint cultivar improvement. Mol Plant. 2017;10:323–39. 27867107 10.1016/j.molp.2016.10.018

[3] Tsai H, Kippes N, Firl A, Lieberman M, Comai L, Henry IM. Efficient construction of a linkage map and haplotypes for *Mentha suaveolens* using sequence capture. G3. 2021;11(9):jkab232. 10.1093/g3journal/jkab232 PMC8496254 34544134

[4] Simão FA, Waterhouse RM, Ioannidis P, Kriventseva EV, Zdobnov EM. BUSCO: assessing genome assembly and annotation completeness with single-copy orthologs. Bioinformatics. 2015;31:3210–2. 26059717 10.1093/bioinformatics/btv351

[5] Seppey M, Manni M, Zdobnov EM. BUSCO: Assessing Genome Assembly and Annotation Completeness. Methods Mol Biol. 2019;1962:227–45. 31020564 10.1007/978-1-4939-9173-0_14

[6] Kim D, Paggi JM, Park C, Bennett C, Salzberg SL. Graph-based genome alignment and genotyping with HISAT2 and HISAT-genotype. Nat Biotechnol. 2019;37:907–15. 31375807 10.1038/s41587-019-0201-4 PMC7605509

[7] Brůna T, Hoff KJ, Lomsadze A, Stanke M, Borodovsky M. BRAKER2: automatic eukaryotic genome annotation with GeneMark-EP + and AUGUSTUS supported by a protein database. NAR Genom Bioinform. 2021;3:lqaa108. 33575650 10.1093/nargab/lqaa108 PMC7787252

[8] Ou S, Su W, Liao Y, Chougule K, Agda JRA, Hellinga AJ, et al. Benchmarking transposable element annotation methods for creation of a streamlined, comprehensive pipeline. Genome Biol. 2019;20:275. 31843001 10.1186/s13059-019-1905-y PMC6913007

[9] Genbank. Chromosome-scale assembly of apple mint (*Mentha suaveolens*). NCBI. 2024. https://identifiers.org/ncbi/insdc.sra:SRP507005 10.1186/s12863-024-01278-y PMC11549836 39516760

[10] FigShare. Chromosome-scale assembly of apple mint (*Mentha suaveolens*). 2024. 10.6084/m9.figshare.26938906.v1 10.1186/s12863-024-01278-y PMC11549836 39516760

[11] GenBank. Genome assembly MenthaSuaveolens85_v8. NCBI. 2024. https://identifiers.org/ncbi/insdc.gca:GCA_041501505.1 [11].

[12] Peters VCT, Dunkel A, Frank O, Rajmohan N, McCormack B, Dowd E, et al. High-throughput flavor analysis and mapping of flavor alterations Induced by different genotypes of Mentha by means of UHPLC-MS/MS. J Agric Food Chem. 2022;70:5668–79. 35475602 10.1021/acs.jafc.2c01689

